# Barriers and facilitators of respiratory syncytial virus (RSV) vaccine confidence among a community-based sample of predominantly Latino older adults in San Francisco

**DOI:** 10.1016/j.jvacx.2026.100824

**Published:** 2026-04-30

**Authors:** Fernanda Amaya, Lisa Geronimo, Sara Colom, Dave Graham-Squire, Noel León, Susana Rojas, Douglas Black, Shalom Bandi, Efrain Barrera, Francisco Herrera, John A. Sauceda, Julia Lechuga, Diane Havlir, Carina Marquez

**Affiliations:** a University of California San Francisco, 2540 23rd St, San Francisco, CA 94110, USA; b Latino Task Force, 701 Alabama St, San Francisco, CA 94110, USA; c Nuevo Sol Day Laborer and Domestic Worker Center San Francisco, 2973 16th St Suite 300, San Francisco, CA 94103, USA

**Keywords:** Vaccine uptake, Latinos, Older adults, RSV, Vaccine knowledge

## Abstract

**Background:**

The new and effective RSV vaccine can reduce morbidity from RSV among older adults, yet uptake is below goal and there are differences across race/ethnicity groups. Thus, we sought to identify barriers and facilitators to RSV vaccine uptake among Latino older adults and evaluate the potential of multi-generational vaccine recommendation.

**Methods:**

In this cross-sectional study, we administered 403 surveys (200 among adults 60+) to a convenience sample of adults at Latino-focused community-based organizations. Surveys assessed barriers and facilitators of RSV vaccination using the Behavioral and Social Drivers (BeSD) framework. We assessed sociodemographic variables and the odds of being motivated to get or recommend the RSV vaccine with a logistic regression, adjusting by age, sex, and race.

**Results:**

Majority of participants were Latino (94.5%) and born outside of the U.S. (87%). Among adults 60+, 3% received the RSV vaccine, whereas 46% received the zoster vaccine and 96% received a COVID vaccine. Prior to survey completion, 60% had never heard of the RSV virus. Once made aware of RSV and vaccine eligibility, 67% were motivated to get vaccinated and 68% felt the vaccine was very important. Older adults' motivation to get the RSV vaccine was linked to confidence in vaccine safety (aOR: 8.9, 95%CI: 2.9–26.9), trust in health workers (aOR: 5, 95%CI: 1.7–14.7), and social norms— family and friends want them to get vaccinated (aOR: 5.2, 95%CI: 2.5–10/8). Among people 18–59 years, 76% discuss health matters with their older family members and 69% would recommend the RSV vaccine.

**Conclusion:**

Though uptake was only 3% at baseline, motivation to get vaccinated was high after hearing about RSV vaccine eligibility. Strategies to increase uptake should include education from trusted sources including health care providers, family members and friends about vaccine eligibility and access.

## 1. Background

The respiratory syncytial virus (RSV) vaccine is a new, safe and effective vaccine [1] that has the potential to reduce the large burden of RSV disease in older adults—which causes an estimated 1.4 million outpatient visits, 159,000 hospitalizations and 12,700 deaths every year [2,3]. Despite its potential to reduce morbidity and mortality, uptake has remained suboptimal since its approval in 2023. As of 2025, only 38% of adults aged 60–74 years and 47.5% of adults over the age of 75 years have been vaccinated nationwide [4]. Disparities in RSV uptake by race/ethnicity, socioeconomic status, and insurance are also emerging. Uptake among Latinos over the age of 75 is lower than non-Latino white people of the same age—29.5% vs. 50.8%, respectively [5]. While barriers to vaccination are shared across different racial/ethnic groups, Latino older adults may face specific barriers, including lack of language concordant vaccine information, experiences of racial discrimination, and structural barriers (e.g., transportation, insurance coverage). The potential public health benefits of this new vaccine will not be realized if uptake remains sub-optimal with disparate uptake across sociodemographic groups in the United States.

Research on other vaccines show clear facilitators of uptake, including the importance of knowledge about vaccine eligibility, access, safety, and health beliefs about susceptibility of infection. Prior data has also shown that social networks and norms can increase vaccination [6,7], yet few have leveraged this for cross-generational vaccine recommendation. Data on other RSV-specific barriers and facilitators of uptake among older adults is also limited. The few studies on RSV uptake highlight low awareness of eligibility and the importance of belief in the safety of the RSV vaccine [8], but data on the challenges and facilitators of RSV vaccination are limited and needed to accelerate uptake and reduce disparities.

This study sought to characterize the behavioral and social drivers of RSV vaccination among Latino older adults living in San Francisco using the WHO Behavioral and Social Drivers of vaccination (BeSD) [9] framework and identify intervention components to increase uptake. Based on our prior work with COVID-19 vaccine uptake [4,10] which showed high vaccine confidence in safety and efficacy as defined by BeSD and highlighted access as the predominant barrier, we hypothesized Latino older adults would have high vaccine confidence in safety and efficacy. Despite high confidence, we hypothesized that uptake would be low due to access-related barriers and knowledge of vaccine availability. Examining RSV vaccine uptake two years after introduction [11] allows a unique opportunity to assess confidence in safety and uptake of a new vaccine in the post-pandemic era in the climate of rising vaccine hesitancy. Our second objective was to assess the potential role of younger adults in recommending the RSV vaccine, as few studies look at the contribution of cross-generational social networks and their impact on vaccine uptake.

## 2. Methods

### 2.1. Study design and setting

In this cross-sectional study, we administered a survey on barriers and facilitators of RSV vaccination to a convenient sample of older adults receiving services at Latino-focused community-based organizations (CBOs). This study was done in collaboration with the San Francisco Latino Task Force, a coalition of over 30 Latino focused CBOs who are also a part of the Unidos en Salud community academic partnership with the University of California, San Francisco. Participants were recruited from Latino focused CBOs and community-based locations: food distribution sites, churches, senior groups, day laborer collectives, and community health and resource hubs. Participants were eligible to complete the survey if they were over the age of 18 and spoke either English or Spanish. We administered the survey until we reached a pre-specified sample size goal of 200 participants The final sample size consisted of 200 participants aged 18 to 59 years and 203 aged 60 years and above. This study received Institutional Review Board approval from the University of California, San Francisco Human Subjects Research Program Protection and participants provided written informed consent prior to participation.

### 2.2. Study procedures and measures

Surveys were conducted through REDCap [12] between November 2024 and February 2025. Participants were compensated $20 via cash or visa gift card upon completion. Surveys could also be completed verbally with the assistance of a bilingual team member, per participants' preference and participants who did not feel comfortable with reading the survey questions or using the technology on their own.

Eligible participants were first asked to self-report their perceived knowledge of RSV followed by a series of sociodemographic and health-related questions, including their vaccination history. Respondents' racial and ethnic identity was determined using the question ‘What is your race or ethnicity? (check all that apply),’ and answers that included the option ‘Latino or Hispanic’ were considered Latino. Respondents who selected ‘American Indian from South or Central America’ were also considered Latino. Questions for participants 60 years of age and older focused on barriers and facilitators to RSV vaccination, using the World Health Organization's Behavioral and Social Drivers of Vaccination standardized questions and toolkit [13]. For participants aged 18–59 years, we adapted the BeSD of vaccination questions to assess barriers and facilitators to recommending RSV vaccination. This was done by modifying references of COVID-19 to reflect RSV. We used the validated Brief Acculturation Scale for Hispanics (BASH) [14] to assess levels of acculturation and defined participants' average scores that were less than or equal to 3 as low and scores that were higher than 3 as high. We also used the validated Health Literacy [15] scale and defined participants scores as having either limited (4–12), marginal (13–16), or adequate (17–20) health literacy.

**Fig. 1.**
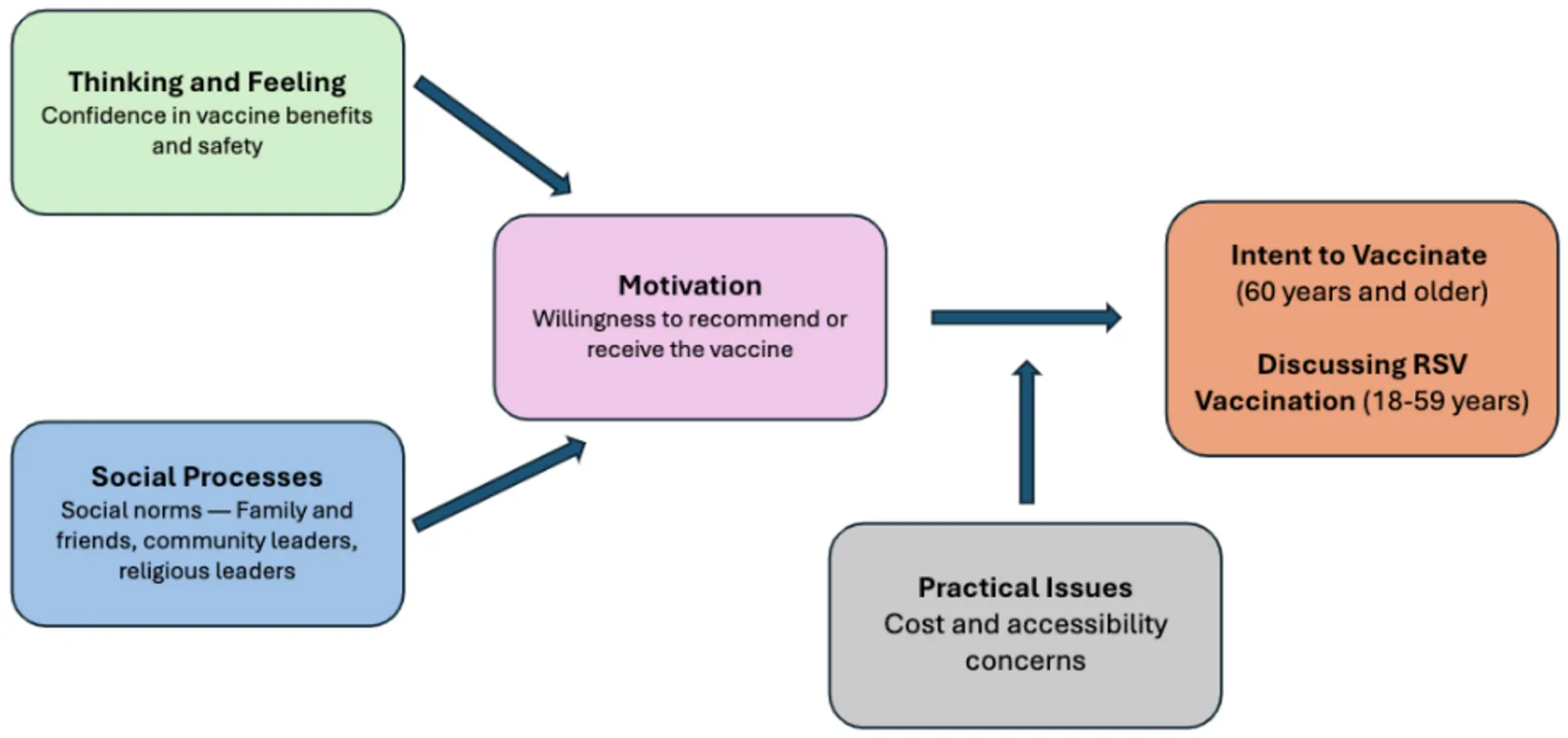
Modified RSV behavioral and social drivers of vaccination (BeSD) framework for respiratory syncytial virus (RSV).

The BeSD framework consists of three stages: confidence, motivation, and behavior, leading to vaccine uptake. Confidence is made up of a person's thinking and feeling, and social processes, which impact one's motivation to get the vaccine. Once one is motivated to get the vaccine, the ability to get the vaccine is affected by practical/access-related barriers (Fig. 1). We then sought to assess predictors of our two dichotomized primary outcomes: (1) RSV vaccine confidence-assessed with the BeSD question ‘How important do you think getting an RSV vaccine will be for your/ your eligible family members’ health?’ and we defined confident as: ‘moderately important,’ or ‘very important.’ Participants' confidence in the RSV vaccine's safety was also collected, but not included as a primary outcome in the final analysis. The second primary outcome, (2) motivation to get the RSV vaccine (participants aged 60 years and above) was determined using the question ‘Do you want to get the RSV vaccine?’ and defined as ‘you want to,’ ‘you do not want to,’ ‘you are not sure,’ or ‘you are already vaccinated.’ Lastly, (2) motivation to recommend the RSV vaccine to friends and family (participants aged 18–59 years) was assessed using the question ‘Would you recommend the RSV vaccine to your friends or family members?’, and defined as ‘yes,’ ‘no,’ ‘maybe,’ or ‘I don't know.’ The responses ‘maybe’ and ‘I don't know’ were offered as distinct choices to reflect different determinants of vaccine decision making. ‘Maybe’ was included to describe a positive willingness or potential to recommend, while ‘I don't know’ was used to reflect a lack of information to form an intention to recommend.

### 2.3. Statistical analysis

Descriptive statistics were calculated for demographic variables, BeSD indicators, and indicators of RSV vaccine confidence and motivation—both overall and stratified by age group (“under 60 years” and “over 60 years”). For continuous variables, the median and interquartile range (IQR) were reported; for categorical variables, frequencies and percentages were presented.

Binary outcome indicators were created for the following constructs: motivation (“Would you recommend the RSV vaccine?” or “Do you want to get the RSV vaccine?”), discussing health matters with family (“Do you discuss health matters with family members?”), and confidence (“How confident are you in the vaccine?”). For motivation, responses were coded as 1 (“yes”) and 0 (“no”, “I don't know”, or “maybe”). For confidence, responses were coded as 1 (“moderately” or “very much”) and 0 (“not at all” or “a little”). Discussing health matters with family did not require regrouping. In all cases, the absence of the desired attribute was coded as 0, and its presence as 1 for downstream analysis.

Multivariable logistic regression models were used to examine associations with each outcome: motivation, discussing health matters with family, and confidence within the ‘under 60 years’ and ‘over 60 years and’ age groups, separately. To evaluate the relationship between each predictor and outcome while accounting for potential multicollinearity and mediation between BeSD domains, a separate multivariable model was conducted for each combination of outcome and primary predictor. These predictors included: income, acculturation (BASH score), U.S.-born status, and indicators of thinking and feeling and social processes. This approach allowed for the identification of key individual predictors while adjusting a priori for race/ethnicity, age, and gender, given their documented associations with vaccine confidence. To account for multiple comparisons, a Bonferroni correction was applied to the *p*-values. All analyses were conducted using R v4.4.2 [16].

## 3. Results

### 3.1. Participant characteristics

Demographics of study participants can be found in Table 1. A total of 403 people participated in the survey with 203 participants aged 18–59 years and 200 aged 60 years and older. Most were Latino (*n* = 381, 94.5%), born outside of the Unites States (*n* = 352, 87.3%), and selected Spanish as their preferred language (*n* = 352, 87.3%). Only 3% of those 60 years or older had received the RSV vaccine, whereas 47% received the zoster vaccine and 96% had received at least one COVID-19 vaccine since its introduction. Most also had both insurance (*n* = 340, 83%) and a primary care provider (*n* = 295, 73%).

### 3.2. RSV Vaccine knowledge and BeSD indicators

In Table 2 we describe the BeSD barriers and facilitators of vaccine uptake. Among adults 60 years and over, 60% had never heard of the RSV vaccine. Once participants were aware of RSV and vaccine eligibility, 67% were motivated to get vaccinated and 86% felt the vaccine was important. Practical challenges were still reported, with 73% of older participants expressing cost concerns.

Adults aged 18–59 years showed similar trends, with 57% of participants having never heard of RSV prior to the survey. Like those 60 years of age and older, most received a COVID-19 booster since becoming available (*n* = 188, 92.6%). This age group's confidence in the vaccine mirrored older adults, with 88.6% feeling that the RSV vaccine was important for the health of their older friends and family. Lastly, we found that 76.6% of younger adults discussed health matters with their older family members, and 69.7% were likely to recommend the RSV vaccine to their eligible friends and family members.

Predictors of RSV Vaccine Confidence, Motivation, and Discussing Health with Family.

Results from the multivariable statistical analysis for adults 60 years and over can be found in Table 3. Vaccine confidence was positively associated with perceived vaccine safety (aOR: 15.1, 95%CI: 4.2–54.7), confidence in health workers (aOR: 11.3, 95%CI: 3.7–34.3), and social norms– family and friends want them to get vaccine (aOR: 6.6, 95%CI: 2.7–15.8), but inversely associated with health literacy (aOR: 0.2, 95% CI: 0.1–0.5) when adjusting for age, sex, and race/ethnicity. Likewise, motivation to get the RSV vaccine was positively associated with confidence in vaccine safety (aOR: 8.9, 95%CI: 2.9–26.9), trust in health workers (aOR: 5, 95%CI: 1.7–14.7), and social norms– family and friends want them to get vaccine (aOR: 5.2, 95%CI: 2.5–10.8). Contrary to our initial expectations, vaccine confidence and motivation to get vaccinated was not found to be associated with older adults' level of acculturation.

Table 4 shows modeling results for adults ages 18–59 years. Like older participants, younger adults' confidence was also found to be related to perceived vaccine safety (aOR: 11.5, 95%CI: 2.3–56.9) and trust in health workers (aOR: 11.1, 95%CI: 3.9–31.4). Moreover, social norms—wanting family and friends (aOR: 12.3, 95%CI: 4.1–37), religious leaders (aOR: 42.8, 95%CI: 7–261.4), and community leaders (aOR: 5.7, 95%CI: 0.7–47.4) to get the vaccine were also found to be linked to younger adults' confidence in the vaccine (Table 4). Younger people's intentions to refer the RSV vaccine followed similar trends, with social norms— family and friends (aOR: 11.8, 95%CI: 5.2–26.7) and religious leaders (aOR: 32, 95%CI: 7.4–138.1) wanting their eligible family to get the vaccine related to willingness to recommend. Finally, perceived vaccine importance (aOR: 7.6, 95%CI: 2.8–20.9), safety (aOR: 10.5, 95%CI: 4–27.7), and trust in health workers (aOR: 3.9, 95%CI: 1.9–8) were found to be linked to younger adults' motivation to recommend the vaccine.

## 4. Discussion

In this community-based sample of predominantly Latinos who were born outside of the United States, knowledge of the RSV virus and uptake of the vaccine were low, with only 3% of older adults receiving the vaccine and only 39% reporting any knowledge of RSV. Despite low uptake and awareness, confidence in the vaccine and motivation to get the RSV vaccine was high among older adults once participants were made aware of the virus and vaccine eligibility. Our data also highlight the potential for leveraging cross-generational networks to increase vaccine uptake, as we found that 76.6% of adults between the ages of 18–50 years discussed health matters with older family members, and 69.7% were highly likely or likely to recommend the RSV vaccine to family members. Interventions to increase knowledge of vaccine awareness from trusted messengers and across social networks have the potential to address the already large gaps in RSV vaccine uptake, which consists of 29.5% vaccine uptake nationwide among Latinos compared to 50.8% among non-Latino/Hispanic white residents as of July 2025 [5].

This study applied BeSD indicators to RSV vaccination and our findings highlight areas for intervention, namely the importance of increasing RSV knowledge (thinking and feeling domain), the potential of leveraging the strong motivation to get the vaccine which likely is due to high confidence in vaccine safety, vaccine efficacy (thinking and feeling domain) and high trust in providers and community or religious leaders (social norms domain). Applying the BeSD framework (Fig. 1) can guide practical interventions to improve vaccine uptake. For example, interventions to increase confidence in vaccine safety and efficacy include expanding language concordant education both in the clinic and in community. Additionally, applying the BeSD framework has the potential to dramatically shift patient awareness in the thinking and feeling domain [10,17], social-network interventions or outreach by trusted religious or community leaders to strengthen social norms [18,19]. Lastly, it emphasizes efforts to address practical barriers such as navigation to pharmacies and providing information about insurance coverage [20].

### 4.1. Thinking and feeling domain

Despite over two years RSV vaccine availability and commercial advertising, information about the vaccine was a key barrier. Recent studies found that in a sample of adults hospitalized for respiratory viruses, nearly half of adult participants reported having never heard of RSV when asked to describe their RSV knowledge [8]. Our findings show an even wider gap among predominantly low-income immigrant Latinos. Latino older adults experience unique barriers and facilitators to vaccine uptake. Among those most commonly cited as obstacles to vaccination, language barriers, geographical inaccessibility, and unaffordability [21] were the most frequent. While the knowledge gap is large, there are many opportunities for action—including language accessible education and outreach that specifically addresses the gap in the Latino community.

Trust in health care providers (HCP) was high, but only 14.6% of eligible adults reported that their HCP discussed RSV Vaccination with them [22]. Provider education and clinic-based interventions targeting providers could be a key intervention in addressing the knowledge gap. A provider's clear recommendation has repeatedly been shown to improve vaccine uptake, and most eligible participants indicated a preference to receive their RSV vaccine at their doctor's office (54.8% of adults 60 years and older). Despite preference to receive the vaccine in clinic, RSV vaccines are often not available in medical offices, due to cost and reimbursement barriers. A study from the CDC showed that 81.7% of adults eligible for the RSV vaccine were vaccinated at a pharmacy or other nonmedical setting, and 63.3% of those vaccinated in nonmedical settings did not have health insurance [22]. Navigating to a pharmacy presents can offer new opportunities but also practical challenges, especially with pharmacy deserts and lower health and tech literacy. Limited in-clinic access to the RSV vaccine may reduce likelihood that health care providers discuss the vaccine during a clinic visit. In San Francisco, the RSV vaccine is not readily available in the clinics within one of the largest public health networks that serves publicly insured patients [23]. This access barrier may make it less likely that health care providers discuss the vaccine in clinic, potentially lowering patient awareness and uptake.

### 4.2. Social norms domain and social networks

Educational interventions outside of brick-and-mortar health facilities from trusted community messengers played a large role in increasing COVID-19 vaccination and conversations surrounding vaccination [4], and our data highlight the importance of this for RSV. Social norms supporting vaccination were high in our sample, and as expected, these were strongly correlated with motivation to get the RSV vaccine and confidence in vaccine effectiveness. Leveraging community trust through trusted messengers from religious and community leaders shows promise for vaccine uptake outside of pandemic settings, especially for those who do not have regular access to a primary care provider [10].

Family and cross-generational social networks show great potential to increase vaccine uptake and could be especially important for those living in multigenerational households. In this study, nearly 77% of Latinos aged 18–59 years discussed health matters with their older family members, and 69% (*n* = 130) anticipated being willing to recommend the vaccine to their eligible family and friends if they felt the confident in the vaccine's benefits. While prior literature highlights the importance of social networks in promoting vaccines from COVID-19 to HPV [24], we contribute to the literature by assessing cross-generational social networks [10].

Few studies characterize predictors of younger generations to recommend vaccination for older adults. Our data shows that discussing health matters with older adults is common, with 76.6% discussing health matters and 69% being more likely to recommend the vaccine if they felt confident in the vaccine's benefits. Previous literature has found that individuals were more likely to have a positive attitude on vaccines, get vaccinated, and perceive the vaccine as effective if discussed or recommended by someone in their social network [6,7]. While these trends have been primarily reported through findings related to HPV, they may also be relevant to RSV and other respiratory viruses.

We found likelihood to recommend vaccination was influenced by similar components as vaccine confidence, such as perceived safety, trust in health workers, and social norms. Likelihood to recommend was also higher among older and non-US born participants. These findings emphasize the need for understanding strategies to activate young US-born Latinos by increasing motivation to recommend the vaccine.

The study had key limitations. We used convenience sampling, with a portion of participants enrolled at community sites that provide health services. We are also limited by sample size in each age strata. Additionally, we recognize the possibility of desirability bias, due to in person survey administration. To minimize this, the team members administering the survey reminded participants that the survey was anonymous, that there is no wrong answer and that participants would be compensated for their time regardless of their answers. The team members also allowed participants to complete the survey independently whenever possible and used standardized tools such as the BeSD scales and BASH questionnaires. Lastly, we recognize that administering the survey during respiratory virus season could have increased participants' confidence in vaccine benefits or motivation to get the vaccine. A key strength of our community-based sample is its representation of populations often missed in clinic or hospital-based studies, including uninsured adults and those without primary care providers, who comprised over a quarter of the adults in this study. Despite some limitations, this study is one of the first to leverage participants' strong community ties and use standardized BeSD indicators to assess barriers and facilitators of vaccination.

The present study tested associations between Latino adults' demographic characteristics, barriers and facilitators of vaccination, and RSV vaccine confidence, motivation to be vaccinated and willingness to recommend the RSV vaccine. While current literature has taken steps towards understanding the relationships between these factors for the general population in the context of other vaccines, there is considerably less research on these links as it pertains to Latinos and RSV, especially Latinos who are low-income and born outside the US. Purposeful and language concordant interventions targeting the improvement of vaccine access and education to Latino immigrant communities is the first step in increasing uptake and reducing disparities. Actionable changes aimed at opening conversations surrounding the RSV vaccine at the provider level and maximizing Latinos' strong community ties by leveraging young people's social networks will be key to closing the gap between RSV knowledge and vaccine uptake.

## Figures and Tables

**Table 1:** Demographics.

	Overall	18–59 years	60+ years
**Age (years)**		***N* = 203**	***N* = 200**
Age Median IQR	59 [36,67]	36 [27.5,48]	67 [64,70]
18–25	38 (9.43%)	38 (18.72%)	
26–39	78 (19.35%)	78 (38.42%)	
40–59	87 (21.59%)	87 (42.86%)	
60–69	137 (34%)		137 (68.5%)
70+	63 (15.63%)		63 (31.5%)
**Race and Ethnicity**			
Latino	381 (94.54%)	188 (92.61%)	193 (96.5%)
Non-Latino	29 (7.2%)	21 (10.35)	8 (4%)
**Sex**			
Man	210 (52.11%)	98 (48.28%)	112 (56%)
			
Woman	186 (46.15%)	99 (48.77%)	87 (43.5%)
Other	7 (1.74%)	6 (1.98%)	1 (0.5%)
**Preferred Language**			
English	51 (12.66%)	44 (21.67%)	7 (3.5%)
Spanish	352 (87.34%)	159 (78.33%)	193 (96.5%)
**Primary Occupation**			
Education Finance, sales, and technology	11 (2.73%)	11 (5.42%)	
Food/beverage	28 (6.95%)	23 (11.33%)	5 (2.5%)
Healthcare	11 (2.73%)	7 (3.45%)	4 (2%)
			
Other	40 (9.93%)	36 (17.73%)	4 (2%)
Retired/homemaker	72 (17.87%)	2 (0.99%)	70 (35%)
Student	19 (4.71%)	19 (9.36%)	
Tradesperson, cleaning, or personal services	94 (23.33%)	33 (16.26%)	61 (30.5%)
Unemployed	128 (31.76%)	72 (35.47%)	56 (28%)
**Household income**			
Less than $3000 a month	286 (70.97%)	119 (58.62%)	167 (83.5%)
			
Between $3000 - $5000 per month	41 (10.17%)	30 (14.78%)	11 (5.5%)
Between $5000 - $8000 per month	10 (2.48%)	10 (4.93%)	
I prefer not to answer	59 (14.64%)	38 (18.72%)	21 (10.5%)
More than $8000 per month	7 (1.74%)	6 (2.96%)	1 (0.5%)
**Primary Care Provider**			
Yes	295 (73.2%)	115 (56.65%)	180 (90%)
No	101 (25.06%)	81 (39.9%)	20 (10%)
I don't know	7 (1.74%)	7 (3.45%)	
**Health Insurance**			
Yes	337 (83.62%)	153 (75.37%)	184 (92%)
No	55 (13.65%)	41 (20.2%)	14 (7%)
I don't know/ Prefer not to disclose	11 (2.73%)	9 (4.44%)	2 (1%)
**Type of Health Insurance**			
Private	54 (13.4%)	37 (18.23%)	17 (8.5%)
Medi-Cal/ Healthy San Francisco	243 (60.3%)	116 (57.14%)	127 (63.5%)
Medicare	43 (10.67%)	2 (0.99%)	41 (20.5%)
I don't know	63 (15.63%)	48 (23.65%)	15 (7.5%)
**Born in the US**			
No	352 (87.34%)	161 (79.31%)	191 (95.5%)
Yes	51 (12.66%)	42 (20.69%)	9 (4.5%)
**Received RSV Vaccine**			***N* = 199**
No			185 (92.96%)
Yes			6 (3.02%)
I don't know			8 (4.02%)
**Received COVID-19 Vaccine**			
No	20 (4.96%)	14 (6.9%)	6 (3%)
Yes	381 (94.54%)	188 (92.61%)	193 (96.5%)
I don't know	2 (0.5%)	1 (0.49%)	1 (0.5%)
**Received Zoster Vaccine**			**N = 199**
No			97 (48.74%)
Yes			93 (46.73%)
I don't know			9 (4.52%)
**Discuss Health with Over 60 Family & Friends (If Under 60)**		***N* = 201**	
No		47 (23.38%)	
Yes		154 (76.62%)	
**Would Recommend the RSV Vaccine (If Under 60)**		**N = 201**	
No		8 (3.98%)	
Yes		140 (69.65%)	
Maybe		30 (14.93%)	
I don't know		23 (11.44%)	
**BASH Score**			
Low	346 (85.86%)	155 (76.35%)	191 (95.5%)
High	57 (14.14%)	48 (23.65%)	9 (4.5%)
**Health Literacy Score**			**N = 199**
Limited			155 (77.89%)
Marginal			44 (22.11%)
**What additional information would you like to know about the RSV Vaccine?**		**N = 201**	
Efficacy	62 (15.38%)	62 (30.85%)	
			
Eligibility	26 (6.45%)	26 (12.94%)	
Safety	20 (4.96%)	20 (9.95%)	
Other	93 (23.07%)	93 (46.27%)	
**Where do you get information about vaccines?**			
Your doctor or health care provider	176 (43.67%)		176 (88%)
Newspapers	101 (25.06%)		101 (50.5%)
Social Media	103 (25.56%)		103 (51.5%)

For Race and Ethnicity, respondents could select more than one option; therefore, percentages may total to more than 100%.

Results shown include frequency and percentages within the sample. Groups with a unique N have been listed.

**Table 2:** Behavioral and Social Drivers of RSV Vaccination Among Adults.

Thinking & Feeling Domain		
BeSD indicator	18–59 years	60+ years
Confidence in Vaccine Benefits (primary measure)		
Not at all important	7 (3.48%)	17 (8.54%)
A little important	16 (7.96%)	11 (5.53%)
Moderately important	36 (17.91%)	34 (17.09%)
Very important	142 (70.65%)	137 (68.84%)
Confidence in RSV Vaccine Safety		
Not at all safe	8 (3.98%)	12 (6.03%)
A little safe	53 (26.37%)	17 (8.54%)
Moderately safe	57 (28.36%)	50 (25.13%)
Very Safe	83 (41.29%)	120 (60.3%)
Confidence in Health Care Workers		
Not at all	9 (4.5%)	7 (3.52%)
A little	39 (19.5%)	10 (5.03%)
Moderately	66 (33%)	61 (30.65%)
Very much	86 (43%)	121 (60.8%)
RSV Vaccine Knowledge		
I have never heard of RSV before	114 (57.29%)	119 (60.41%)
I knew very little information about RSV	51 (25.63%)	57 (28.93%)
I knew some information about RSV	21 (10.55%)	16 (8.12%)
I knew a lot of information about RSV	13 (6.53%)	5 (2.54%)
Social Processes Domain		
Family Norms: Family & Friends Want You to Get RSV Vaccine		
No	46 (22.89%)	47 (23.62%)
Yes	155 (77.11%)	152 (76.38%)
Social Norms: Religious Leaders Want You to Get RSV Vaccine		
No	19 (9.45%)	15 (7.54%)
Yes	70 (34.83%)	46 (23.12%)
Not applicable	25 (12.44%)	30 (15.08%)
I don't know	87 (43.28%)	108 (54.27%)
Social Norms: Community Leaders Want You* to Get RSV Vaccine		
No	5 (2.49%)	16 (8.04%)
Yes	120 (59.7%)	66 (33.17%)
I don't know	76 (37.81%)	117 (58.79%)
Healthcare Worker Recommendations		
No	96 (47.76%)	155 (77.89%)
Yes	68 (33.83%)	29 (14.57%)
I don't know	37 (18.41%)	15 (7.54%)
Motivation		
Intent to Get Vaccine: Do you want to get the RSV vaccine?		
You are already vaccinated	13 (6.47%)	8 (4.02%)
You are not sure	75 (37.31%)	39 (19.6%)
You do not want to	12 (5.97%)	18 (9.05%)
You want to	101 (50.25%)	134 (67.34%)
Practical Issues		
Know Where: Where Would You Get the RSV Vaccine?		
I do not want to get it		7 (3.52%)
I don't know where to get it	57 (28.36%)	15 (7.54%)
My doctor's office	123 (61.19%)	109 (54.77%)
The pharmacy	21 (10.45%)	68 (34.17%)
Affordability: How Easy is it for you to pay for the RSV Vaccine?		
Not at all easy	77 (38.31%)	145 (72.86%)
A little easy	46 (22.89%)	8 (4.02%)
Moderately easy	42 (20.9%)	8 (4.02%)
Very Easy	36 (17.91%)	38 (19.1%)
Received Vaccine		
Have you received the RSV Vaccine?		
No		185 (92.96%)
Yes		6 (3.02%)
I don't know		8 (4.02%)

Results shown include frequency and percentages within each sample.

**Table 3:** Predictors of Intention to Vaccinate (BeSD domain: Motivation) and Confidence in Vaccine Benefits (BeSD Confidence) Among Adults 60 Years of Age and Older.

	Outcome: Motivation (BeSD Indicator)- Do you want to get the RSV Vaccine?	Outcome: Confidence (BeSD Indicator) - How confident are you in the RSV Vaccine?
Predictors	aOR (95% CI)	aOR (95% CI)
Demographics		
**Age (Ref: 60–70 years)**		
70+ years	0.6 (0.3–1.2)	0.4 (0.2–1)
**Gender: Woman (Ref: Man)**	1.8 (0.9–3.4)	1.3 (0.6–3)
**Ethnicity: Latino (Ref: Non-Latino)**	4.6 (0.8–26.3)	2.4 (0.4–14.1)
**Income (Ref: Less than $3000 a month)**		
Between $3000–5000 a month	1.2 (0.3–4.8)	1.8 (0.2–15.3)
**BASH Score (Ref: Low)**		
High	1.1 (0.2–6.7)	0.3 (0–1.8)
**Born in US (Ref: Not born in US)**	1 (0.2–5.2)	1.7 (0.2–17.5)
**Health Literacy Scale (Ref: High)**		
Marginal	0.4 (0.2–0.8)	0.2 (0.1–0.5)**
**Thinking and Feeling Domain**		
**Confidence in Vaccine effectiveness (Ref: Not at all to A Little Important)**		
Moderately Important	37.8 (7.1–201.8)**	
Very Important	76.6 (16.4–357.3)**	
**Confidence: Vaccine Safety (Ref: Not Safe)**		
Moderately Safe	8.9 (2.9–26.9)**	15.1 (4.2–54.7)**
**Confidence in Health Workers (Ref: Not At All to A Little)**		
Moderate to Very Much	5 (1.7–14.7)*	11.3 (3.7–34.3)**
**Social Processes**		
**Social Norms: Family and Friends (Ref: No)**	5.2 (2.5–10.8)**	6.6 (2.7–15.8)**
**Social Norms: Religious Leaders (Ref: No)**		
**Social Norms: Community Leaders (Ref: No)**	1.4 (0.5–4.6)	1.7 (0.5–5.9)
**Provider Trust: How much do you trust the health workers who give the vaccine:**	5 (1.7–14.7)*	11.3 (3.7–34.3)**

* *p* < .05

** *p* < .01.

For each outcome, separate models were fit for each predictor listed row-wise, each adjusting for age, race, and gender. All p-values are Bonferroni adjusted, with raw p-values multiplied by 12 for the 12 comparisons. Cells are blank where sample sizes within predictor strata were insufficient to support contrast estimation.

**Table 4:** Predictors of Willingness to Recommend the Vaccine (BeSD: Recommending the Vaccine) and Confidence in Vaccine Benefits (BeSD: Confidence) Among Adults 18–59 Years of Age.

	Outcome: Motivation (BeSD Indicator)- Would you recommend the RSV vaccine?	Outcome: Discussing Health Matters with Family	Outcome: Confidence (BeSD Indicator)- How confident are you in the vaccine?
Predictors	aOR (95% CI)	aOR (95% CI)	aOR (95% CI)
Demographics			
**Age category (Ref: 18–25 years)**		
26–39 years	1.4 (0.6–3.4)	1.5 (0.6–3.5)	1.4 (0.5–4.3)
40–59 years	1.8 (0.8–4.2)	2.7 (1.1–6.8)	2.8 (0.8–9.5)
**Gender: Woman (Ref: Man)**	0.6 (0.3–1.1)	1 (0.5–2)	2.4 (0.9–6.3)
**Ethnicity: Latino (Ref: Non-Latino)**	0.8 (0.2–3.4)	1.2 (0.3–5.1)	0.9 (0.1–8)
**Income (Ref: Less than $3000 a month)**		
Between $3000–5000 a month	0.7 (0.3–1.7)	0.8 (0.3–2.1)	1.5 (0.3–7.8)
Between $5000–8000 a month	0.8 (0.2–3.4)	2.7 (0.3–23.3)	1.1 (0.1–10.7)
**BASH Score: High (Ref: Low)**	0.8 (0.3–1.7)	0.5 (0.2–1.1)	0.8 (0.3–2.5)
**Born in US (Ref: Not born in US)**	0.8 (0.3–1.7)	0.5 (0.2–1)	0.6 (0.2–1.9)
**Thinking and Feeling Domain**		
**Confidence in Vaccine Benefits (Ref: Not at all important to A Little Important)**		
Moderately Important	1.5 (0.5–4.7)	0.7 (0.2–2.5)	
Very important	7.6 (2.8–20.9)**	1.7 (0.6–4.9)	
**Confidence: Vaccine Safety (Ref: Not Safe)**		
Moderately Safe	10.5 (4–27.7)**	1.8 (0.7–4.4)	11.5 (2.3–56.9)*
**Confidence in Health Workers (Ref: Not at all to a little)**		
Moderate to Very Much	3.9 (1.9–8)	0.8 (0.3–1.8)	11.1 (3.9–31.4)**
**Social Processes**			
**Social Norms: Family and Friends (Ref: No)**	11.8 (5.2–26.7**	1.8 (0.8–4)	12.3 (4.1–37)**
**Social Norms: Religious Leaders (Ref: No)**	32 (7.4–138.1)**	3.2 (0.9–11)	42.8 (7–261.4)**
**Social Norms: Community Leaders (Ref: No)**			5.7 (0.7–47.4)
**Provider Trust: How much do you trust the health workers who give the vaccine**		
Moderate to Very Much	3.9 (1.9–8)	0.8 (0.3–1.8)	11.1 (3.9–31.4)**

* p < .05

** p < .01.

For each outcome, separate models were fit for each predictor listed row-wise, each adjusting for age, race, and gender. All p-values are Bonferroni adjusted, with raw p-values multiplied by 11 for the 11 comparisons. Cells are blank where sample sizes within predictor strata were insufficient to support contrast estimation.

## Data Availability

The data that has been used is confidential.

## Appendix A. Supplementary data

Supplementary data to this article can be found online at https://doi.org/10.1016/j.jvacx.2026.100824.
